# CD23^+^/CD21^hi ^B-cell translocation and ipsilateral lymph node collapse is associated with asymmetric arthritic flare in TNF-Tg mice

**DOI:** 10.1186/ar3452

**Published:** 2011-08-31

**Authors:** Jie Li, Quan Zhou, Ronald W Wood, Igor Kuzin, Andrea Bottaro, Christopher T Ritchlin, Lianping Xing, Edward M Schwarz

**Affiliations:** 1 Center for Musculoskeletal Research, University of Rochester School of Medicine and Dentistry, 601 Elmwood Avenue, Rochester, NY 14642, USA; 2 Department of Microbiology and Immunology, University of Rochester School of Medicine and Dentistry, 601 Elmwood Avenue, Rochester, NY 14642, USA; 3 Department of Pathology and Laboratory Medicine, University of Rochester School of Medicine and Dentistry, 601 Elmwood Avenue, Rochester, NY 14642, USA; 4 Department of Obstetrics and Gynecology, University of Rochester School of Medicine and Dentistry, 601 Elmwood Avenue, Rochester, NY 14642, USA; 5 Department of Urology, University of Rochester School of Medicine and Dentistry, 601 Elmwood Avenue, Rochester, NY 14642, USA; 6 Division of Allergy, Immunology, Rheumatology, Department of Medicine, University of Rochester School of Medicine and Dentistry, 601 Elmwood Avenue, Rochester, NY 14642, USA

## Abstract

**Introduction:**

Rheumatoid arthritis (RA) is a chronic autoimmune disease with episodic flares in affected joints. However, how arthritic flare occurs only in select joints during a systemic autoimmune disease remains an enigma. To better understand these observations, we developed longitudinal imaging outcomes of synovitis and lymphatic flow in mouse models of RA, and identified that asymmetric knee flare is associated with ipsilateral popliteal lymph node (PLN) collapse and the translocation of CD23^+^/CD21^hi ^B-cells (B-in) into the paracortical sinus space of the node. In order to understand the relationship between this B-in translocation and lymph drainage from flaring joints, we tested the hypothesis that asymmetric tumor necrosis factor (TNF)-induced knee arthritis is associated with ipsilateral PLN and iliac lymph node (ILN) collapse, B-in translocation, and decreased afferent lymphatic flow.

**Methods:**

TNF transgenic (Tg) mice with asymmetric knee arthritis were identified by contrast-enhanced (CE) magnetic resonance imaging (MRI), and PLN were phenotyped as "expanding" or "collapsed" using LNcap threshold = 30 (Arbitrary Unit (AU)). Inflammatory-erosive arthritis was confirmed by histology. Afferent lymphatic flow to PLN and ILN was quantified by near infrared imaging of injected indocyanine green (NIR-ICG). The B-in population in PLN and ILN was assessed by immunohistochemistry (IHC) and flow cytometry. Linear regression analyses of ipsilateral knee synovial volume and afferent lymphatic flow to PLN and ILN were performed.

**Results:**

Afferent lymph flow to collapsed nodes was significantly lower (*P *< 0.05) than flow to expanding nodes by NIR-ICG imaging, and this occurred ipsilaterally. While both collapsed and expanding PLN and ILN had a significant increase (*P *< 0.05) of B-in compared to wild type (WT) and pre-arthritic TNF-Tg nodes, B-in of expanding lymph nodes (LN) resided in follicular areas while B-in of collapsed LN were present within LYVE-1+ lymphatic vessels. A significant correlation (*P *< 0.002) was noted in afferent lymphatic flow between ipsilateral PLN and ILN during knee synovitis.

**Conclusions:**

Asymmetric knee arthritis in TNF-Tg mice occurs simultaneously with ipsilateral PLN and ILN collapse. This is likely due to translocation of the expanded B-in population to the lumen of the lymphatic vessels, resulting in a dramatic decrease in afferent lymphatic flow. PLN collapse phenotype can serve as a new biomarker of knee flare.

## Introduction

One of the most intriguing features of rheumatoid arthritis (RA) is the fluctuating disease activity characterized by disease flares and quiescence observed in most patients over time [1,2]. Indeed, despite the advances in treatment over the last decade, control of disease flare remains a major challenge in rheumatology practice [3,4]. The factors responsible for the cyclical exacerbation of joint inflammation are poorly understood, but environmental factors such as pregnancy, changes in weather, stress, smoking and infection have received attention as potential triggers [5-8]. In contrast, relatively little attention has been directed towards the possible role of local factors in RA flare. The fact that an RA flare often occurs asymmetrically in the setting of systemic immune mediated inflammation suggests that events in and around the joint may be of central importance akin to the interplay of osteitis and regional biomechanical forces that lead to enthesopathy in spondyloarthritis [9].

One potential key variable in the development of arthritic flare is regional efferent lymphatic flow from RA joints. It has been known for almost 75 years that lymphatic vessels proliferate at sites of inflammation [10], but the contribution of lymph clearance has been largely overlooked until recently [11]. Studies show that lymphatic clearance serves as a compensatory mechanism to mobilize and transport cells, interstitial fluid and catabolic factors produced during a chronic inflammatory response [12,13]. It is important not to overlook previous observations on rheumatoid lymphedema [14], and classic clinical studies that demonstrated the efficacy of thoracic duct drainage on lymphocyte populations and reduction of clinical symptoms in RA [15]. However, a major obstruction to progress in this field has been the lack of quantitative measures of lymphatic flow. Although case reports with lymphoscintigraphy have posited that patients with tenosynovial inflammation and normal lymphatic drainage demonstrate improved pharmacologic responses and improved clinical outcomes compared to patients with chronic lymphatic vascular damage and persistent oedema [16,17], this theory has yet to be tested in animal models or clinical trials.

Lymphatic research performed on animal models provides a novel opportunity to systematically examine the natural history of inflammatory-erosive arthritis. For example, the critical role of vascular endothelial growth factor C (VEGF-C) and its receptor VEGFR-3 in the formation of new lymphatic vessels (lymphangiogenesis) opened new avenues of research [18,19]; and the dramatic changes in the pulse of efferent lymphatic vessels during the acute (five pulses per minute) and chronic (one pulse per minute) phases of the inflammatory arthritis emphasized the contribution of local variables that had previously been largely unknown [20]. Of critical importance from a translational perspective is the development of novel therapies (for example, flavonoids, VEGF-C) that specifically target lymphangiogenesis and increase lymphatic flow [21]. Equally important is the availability of new methods to assess lymph node (LN) draining function and lymphatic flow *in vivo*, which have the potential to serve as biomarkers of arthritic flare and response to therapy.

CE-MRI is of particular interest because it takes advantage of the redistribution of intravenously delivered gadolinium (Gd-DTPA) to the open sinus spaces of LN [22]. For a readily accessible LN like the popliteal (PLN), CE-MRI can be used to quantify volume (LNvol), the difference between pre and post contrast enhancement (LNCE), and their product (LNcap), which is an estimate of the node's draining capacity [22]. Real time indocyanine green near-infrared (ICG-NIR) lymphatic imaging, a clinically validated approach to map sentinel lymph nodes during tumor resection [23], has been used to quantify various parameters of lymphatic flow over a 1 hr study period and the residual ICG at the injection site 24 hr later [19,20,24].

We applied these longitudinal outcome measures to study the natural history of inflammatory-erosive arthritis in the TNF-Tg [25] and K/BxN [26] murine models of RA, and noted several observations about PLN behavior in relation to the development of ankle and knee synovitis in the animals [19,20,22,27-29]. The PLN displays a significant increase in volume (LNvol; from < 2 to > 10 mm^3^), contrast enhancement (LNCE; from approximately 2 to approximately 4 AU) and capacitance (LNcap; from < 3 to > 40 AU), prior to disease onset, which continues during ankle arthritis in TNF-Tg mice from two to nine months of age [22]. A similar PLN behavior is seen in K/BxN mice as they develop inflammatory arthritis [20], despite the distinct pathologies and triggering events in these two models (tenosynovitis in TNF-Tg [30], versus Fc-receptor and complement activation in K/BxN [31]). Flow cytometry and histology analyses confirmed that the increased volume results from accumulation of lymphatic fluid, associated with the expression of LYVE1+, a lymph specific hylauronic acid receptor, on the surface of lymphatic vessels, and the influx of a unique subset of CD23^+^/CD21^hi ^B cells in inflamed nodes (B-in) [19,20,22,27-29]. Based on these dynamic volume fluxes, we refer to these nodes as "expanding" PLN.

Synovitis with focal erosions in the knees of these animals typically occurs several months after onset of ankle arthritis, and is concomitant with a significant decrease in PLN LNvol, LNCE and LNcap [22,29], which we refer to as "collapsed" PLN. Moreover, arthritic flare in the knee and variations in PLN volumes often occur asymmetrically in the same animal, and some TNF-Tg mice never (> 1 yr) develop knee arthritis in lower limbs that sustain expanding PLN, although they all have severe ankle arthritis [22,29]. These findings indicate that systemic effects such as autoimmunity or aging alone are insufficient to trigger knee flare, and raise the possibility that local factors maybe important. Of note was that flow cytometry of PLN confirmed that the decreased volume is due to the loss of fluid, because no significant difference in cell numbers were detected in the "collapsing" vs. "expanding" PLN [29]. We also failed to detect any significant difference in the B-in population in terms of cell numbers, gene expression and markers of activation and proliferation [29]. Thus, B-in are not an indicator of collapsed PLN and subsequent knee flare, whose mechanism remains unknown. However, detailed immunohistochemistry (IHC) studies revealed that the B-in population translocates from the follicular areas in the expanding PLN, to the paracortical lymphatic sinuses of the collapsed PLN [29]. Additionally, B cell depletion therapy with anti-CD20 antibodies sustained high LNCE of PLN, and anti-CD20 antibody treated TNF-Tg mice did not display asymmetric arthritis similar to the arthritic flare observed in the placebo group [29]. Collectively, these results suggest that: i) expanding PLN protect the adjacent knee from arthritis; and ii) the loss of lymphatic drainage due to PLN collapse precipitates the accumulation of inflammation in the afferent joint that manifests as an arthritic flare. Interestingly, there have been no published studies that specifically focus on lymphatic drainage of the knee. Although some work has been done in this field [32,33], it remains unclear if lymph from the synovium drains directly to PLN or ILN in mice. Here we examined the association of asymmetric TNF-induced knee arthritis with: i) ipsilateral PLN and ILN collapse, ii) translocation of CD23^+^/CD21^hi ^B cells (B-in), and iii) decreased afferent lymphatic flow from the lower limb.

## Materials and methods

### Animals

The 3,647 line of TNF-transgenic mice in a C57BL/6 background were originally obtained from Dr. George Kollias (Institute of Immunology, Alexander Fleming Biomedical Sciences Research Center, Vari, Greece). The TNF-Tg mice are maintained as heterozygotes, such that non-transgenic littermates are used as aged-matched wild type (WT) controls. All animal studies were performed under protocols approved by the University of Rochester Committee for Animal Resources.

### CE-MRI and MR data analysis

Two cohorts of gender mixed TNF-Tg mice at different ages were studied. The first (young) cohort was identified by studying three-month-old TNF-Tg mice (*n *= 10) with frank ankle to ensure disease initiation. At this stage, TNF-Tg mice do not have knee arthritis yet, thus they were longitudinally assessed to capture the initial knee flare These animals received bilateral CE-MRI of their lower limbs every two weeks until asymmetric PLN collapse was detected as previously described [29]. To examine the relationship between knee synovial volume and lymphatic flow to the PLN and ILN, a second cohort of TNF-Tg mice (*n *= 12) with a broad range of arthritis severity (knee synovial volume range of < 1 to > 6 mm^3^) was obtained by studying animals from three to more than seven months of age by CE-MRI. Briefly, anesthetized mice were positioned with their knee inserted into a customized knee coil, and MR images were obtained on a 3 Tesla Siemens Trio MRI (Siemens Medical Solutions, Erlangen, Germany). Amira (TGS Unit, Mercury Computer Systems, San Diego, CA, USA) was used for segmentation and quantification of ankle synovial volume, knee synovial volume, LNvol, LNCE and LNcap as previously described [22,28].

### Histology and Immunohistochemistry

Knee joints were fixed in 4.5% phosphate-buffered formalin and decalcified in 14% EDTA for seven days. Histology sections were stained with Orange G and Alcian Blue (OG/AB) or for tartrate-resistant acid phosphatase as previously described [34]. LNs were processed using two different protocols. For multicolor immunofluorescence microscopy, fresh frozen LNs were cut into 6-μm-thick sections. PLN sections were fixed with 4% paraformaldehyde, rehydrated in PBS, and blocked with rat serum, prior to incubation with PE-conjugated anti-IgM (clone II/41; eBioscience, San Diego, CA, USA) and anti-LYVE-1 (ab14917, Abcam, Cambridge, MA, USA) together with secondary antibody FITC-anti-rabbit IgG (Invitrogen, Carlsbad, CA, USA) or PE-anti-rabbit IgG (Invitrogen). To assess co-localization of B cells within lymphatic vessels, IHC photographs were taken and then analyzed by dividing the number of yellow pixels by the total number of pixels in the manually segmented LN to determine the % of overlapping red and green pixels (Image-Pro Plus Version 5.4.0.2.9 (Media Cybernetics, Inc. Bethesda, MD, USA)). For standard histology, fresh frozen PLN sections were directly used for hematoxylin and eosin (H&E) staining.

### Flow cytometry

A separate cohort of mice was used to quantify the B-in population in PLN and IL from WT, one- to two-month-old TNF-Tg mice (pre-expanding PLN stage) and older TNF-Tg mice with established disease (expanding and collapsed PLN) via multicolor flow cytometry as previously described [29]. Briefly, single-cell suspensions were incubated with a combination of the following fluorochrome-labeled Abs: APC-Alexa 750 anti-B220 (clone RA3-6B2; eBioscience); PE anti-IgM (clone II/41; eBioscience); Pacific Blue anti-CD21/35 (clone 7E9; BioLegend, San Diego, California); and PE-Cy7 anti-CD23 (clone B3B4; BioLegend). Samples were run on an LSRII cytometer and analyzed by FlowJo software (BD Pharmingen, San Diego, California). To control for nonspecific Ab binding, isotype control experiments were conducted and resulted in nonsignificant background stains. To quantify the B-in population, an initial gating on the B220+/IgM+ population was performed. The cells within this gate were analyzed for CD21 and CD23 expression

### Indocyanine green near-IR (ICG-NIR) lymphatic imaging

Lymphatic drainage was quantified by ICG-NIR using a Spy1000 system (Novadaq Technologies, Bonita Springs, Florida) as previously described [20]. The video outputs of the camera were attached to the network (Axis 241SA video server, Lund, Sweden); the image streams were captured (Security Spy by Ben Bird) as QuickTime movies (Apple Computers, Cupertino, California). Individual JPEG image sequences were then exported for further analysis with ImageJ. Indocyanine green (Acorn) was dissolved in distilled water at 0.1 μg/μl, and 6 μl of the green solution was injected intradermally into the mouse footpad or knee joint using a 30-gauge needle. ICG-NIR imaging was performed for 1 hour immediately after ICG injection, and again for 5 minutes 24 hours later. Sequential images from the movie file were exported, and the ICG fluorescence intensity of the injection site and PLN was determined using Image J software (Developed by National Institutes of Health, Bethesda, Maryland) to quantify: i) T-initial (T-in), which is the time it takes for the injected ICG to be detected in lymphatic vessels in the leg; ii) S-max, which is the maximum ICG signal intensity observed in PLN during the first hour imaging session; iii) T-max, which is the time it takes for the PLN to achieve S-max; and iv) percent clearance, which is an assessment of ICG washout through the lymphatics and is quantified as the percent difference in ICG signal intensity at the injection site immediately after administration and 24 hours later. To quantify lymphatic draining in ILN, 6 μl of ICG solution was injected intraarticularly into the knee cavity. Ten minutes after injection the mouse was euthanized, dissected to expose ILNs, and the signal intensity (SI) of the node was determined using Image J.

### Statistical analysis

Two-tailed t-tests were used to make comparisons between groups. Correlations between measures were estimated using Pearson's correlation coefficient and tested for significance using a two-sided t-test test. *P*-values less than 0.05 were considered significant and *P*-values less than 0.01 were considered highly significant.

## Results

### Afferent lymphatic flow to collapsed PLN is significantly decreased compared to expanding PLN

**Figure 1:**
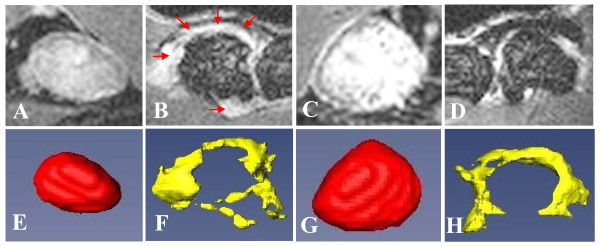
**CE-MRI phenotyping of collapsed vs. expanding PLN and asymmetric knee arthritis in TNF-Tg mice**. TNF-Tg mice (*n *= 10; 20 legs) with ankle arthritis were monitored by CE-MRI to phenotype their PLN as expanding or collapsed. The quantitative data are presented in Table 1. 2D CE-MRI (**A-D**), and 3D reconstructed volumes (**E-H**), of the left (A, B, E, F) and right (C, D, G, H) legs of a representative TNF-Tg mouse with asymmetric arthritis are presented to illustrate the phenotypic differences between collapsed PLN (A, E), which are smaller and have limited contrast enhancement vs. expanding PLN (C, G), which are larger and have saturated contrast enhancement throughout most of the node. The asymmetric arthritic phenotype in this animal is also apparent from the contrast enhancing pannus tissue that surrounds the femoral chondyles in only one knee (red arrows in B), and the larger synovial volume 5.6 mm^3 ^(F) vs. 3.8 mm^3 ^(H).

TNF-Tg mice (*n *= 10) with asymmetric knee arthritis were identified by CE-MRI as previously described [29], and the data are presented in Table 1. Figure 1 is also presented to illustrate the dramatically different phenotypes of expanding vs. collapsed PLN and synovitis in the adjacent knee, as assessed by the primary CE-MRI and 3D reconstructed images. Although the phenotype of the PLN could be subjectively determined by gross assessment of the images, analysis of the CE-MRI data presented in Table 1 revealed non-overlapping threshold values (LN CE = 5 AU and LNcap = 30 AU) that were subsequently used as objective criteria to define the PLN as expanding or collapsed. Based on these criteria, we found that the synovial volume of the knees adjacent to collapsed PLN is significantly greater than expanding PLN (Table 1).

**Table 1:** Expanding vs. collapsed PLN

*Parameter*	*Expanding*	*Collapsed*
LN volume range (mm^3^)	5.0 to 12.7	3.6 to 7.7
LN CE range (arbitrary units)	5.7 to 8.2	2.1 to 4.3
LN capacity range (arbitrary units)	30.5 to 83.5	8.4 to 27.5
Knee synovitis volume (mm^3^)	3.5 ± 0.7	5.5 ± 1.2*
LN CE (arbitrary units)	6.8 ± 0.8	3.4 ± 0.7*
LN capacity (arbitrary units)	52.9 ± 19.1	18.6 ± 6.8*

CE, contrast enhancement; LN, lymph node; PLN, popliteal lymph node. Values are mean ± SD, *n *= 10 for each group. *P < 0.05 vs. expanding.

**Figure 2:**
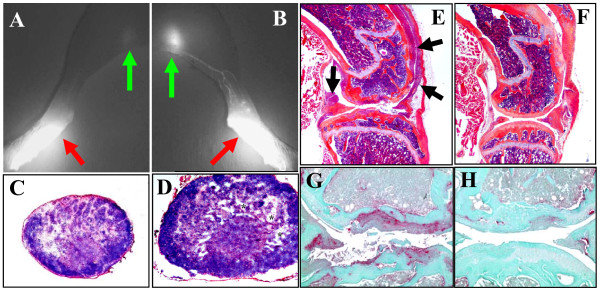
**Asymmetric TNF-induced knee arthritis is associated with ipsilateral PLN collapse and decreased afferent lymphatic flow**. The mice described in Figure 1 were subjected to NIR-ICG imaging to quantify lymphatic drainage from their lower limbs, prior to sacrifice for histology, and data from a representative animal are shown. The NIR-ICG images of the left (**A**) and right (**B**) lower limb of the mouse obtained 30 minutes after the ICG injection into the footpad (red arrows) illustrates the dramatic difference in afferent lymphatic flow to the PLN (green arrows) as evidenced by the lack of signal in the collapsed (A) versus the bright signal in the expanding (B) PLN. Micrographs (5x) of the H&E stained histology of the PLN reveal the shrunken phenotype of the collapsed PLN (**C**), compared to the expanding PLN with enlarged paracortical sinuses (* in **D**). Micrographs of the H&E (**E, F**) and TRAP (**G, H**) stained histology of the knees taken at 5x and 10x respectively, confirmed the presence of extensive synovitis (arrows in E) and focal erosions (G) in the left knee ipsilateral to the collapsed PLN, in contrast to the very early stage arthritis observed in the right knee ipsilateral to the expanding PLN (F, H).

**Figure 3:**
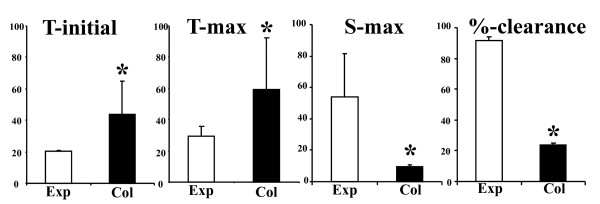
**Afferent lymphatic drainage in the lower limb is significantly decreased after PLN collapse**. NIR-ICG imaging was performed on the TNF-Tg mice described in Figure 1 to quantify lymphatic flow in the lower limb. The real time video of the NIR-ICG imaging session was used to quantify the four outcome measures of lymphatic flow from the foot to expanding (Exp) and collapsed (Col) PLN, and the data are presented as the mean +/- SD for the group (*n *= 3, * *P *< 0.05 vs. Exp).

To further confirm the association of asymmetric lymphatic defects and arthritic flare in TNF-Tg knees with collapsed PLN vs. expanding PLN, we performed ICG-NIR imaging and subsequent histological analyses as illustrated in Figure 2. The ICG-NIR results demonstrated that lymph flow to collapsed PLN is significantly decreased in all of the parameters tested (Figure 3). Histology of the knees of these mice confirmed that advanced inflammatory-erosive arthritis was only present in joints adjacent to collapsed PLN, and that there was little or no evidence of arthritis in the knees adjacent to expanding PLN (Figure 2). Collectively, the findings suggest that the volume of the draining lymph node may be an important variable in the onset of an arthritic flare.

### Lymph from the knee joint primarily drains to ILN

**Figure 4:**
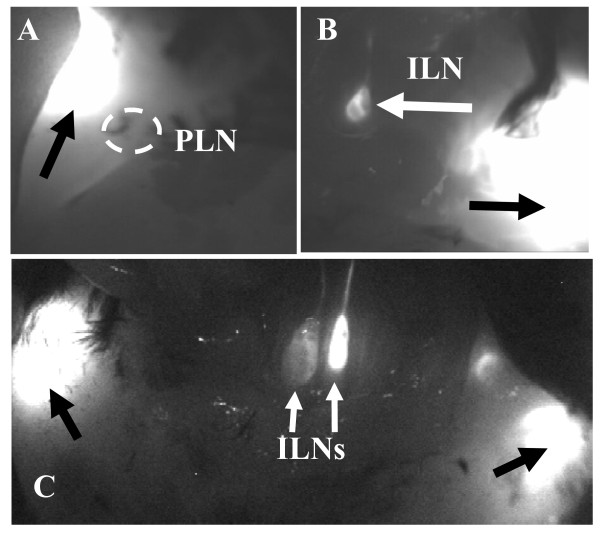
**Ipsilateral ILN drains ICG from WT/pre-arthritic knees not from arthritic knees with collapsed PLN**. Non-recovery ICG-NIR imaging of PLN and ILN was performed on WT mice (*n *= 2) following an intraarticular ICG injection into the knee, in which their abdominal cavity was opened to expose the ILN. NIR-ICG images obtained 10 minutes after injection from one of the animals are shown highlighting the ICG drainage from the injection site (black arrows) to the PLN (**A **dorsal view) versus the ILN (**B **ventral view). Note the absence of ICG signal in the PLN (circled region), and its presence in the ILN (white arrow). Similar non-recovery ICG-NIR imaging was performed on TNF-Tg mice (*n *= 8) with asymmetric knee arthritis, and a ventral view image of a representative animal with collapsed (left) and expanding (right) PLN is shown (**C**). Black arrows indicate the ICG injection site in the knee, and white arrows point to the ILN. Note that ICG has migrated to the right ILN but not the left ILN resulting in a dramatic difference in SI (179 vs. 253).

To directly address the issue of primary efferent lymphatic drainage from the knee joint, we injected ICG intra-articularly into the knee cavity of WT mice, and monitored particle migration by whole body NIR imaging (Figure 4). The results demonstrated that most of the migrating ICG resided in ILN 30 minutes after injection, while there was no detectable signal in PLN at this time. Therefore, since both ILN and PLN drain the lower limb, the most likely explanation for the coincidence between PLN collapse and knee flare is that ipsilateral PLN and ILN collapse occurs simultaneously through some unknown limb-specific mechanism.

### B-in expansion in ILN is similar to that in ipsilateral PLN

**Figure 5:**
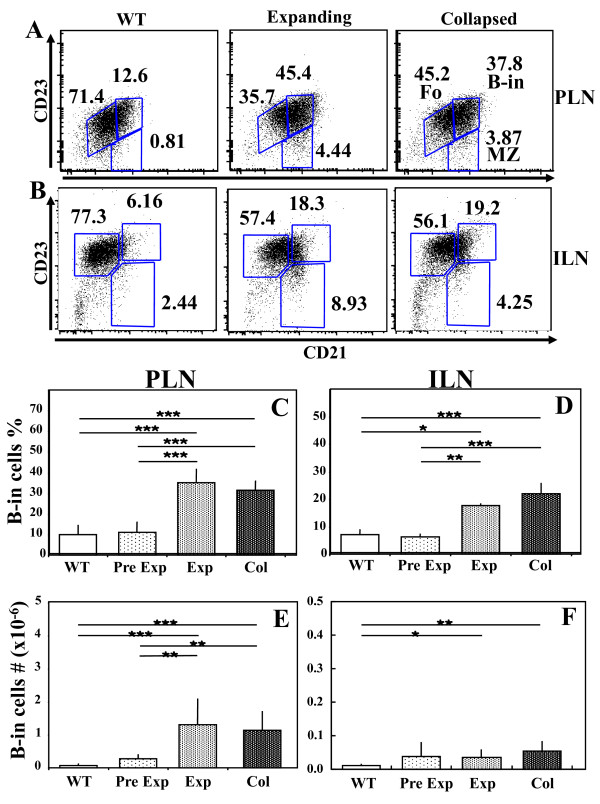
**B-in expansion in Ipsilateral PLN and ILN of TNF-Tg mice with inflammatory arthritis**. PLN and ILN (n ≥ 4) were harvested from wild-type (WT) mice, one- to two-month old TNF-Tg mice before the onset of ankle arthritis and PLN expansion (Pre Exp), and older TNF-Tg mice with established disease after PLN expansion (Exp), or after PLN collapse (Col), and used for multicolor flow cytometry as described in Materials and methods. The B-in population was quantified from the B220+/IgM+ fraction based on CD21 and CD23 staining, as illustrated by representative histograms of ipsilateral PLN (**A**), and ILN (**B**), from each group. This gating approach segregates the phenotypic follicular B cells (Fo), the marginal zone B cells (MZ), and the B-in population. The percentage of each population is shown. The percentage of B-in cells within this B220+/IgM+ fraction from PLN (**C**), and ILN (**D**); and the absolute number of B-in cells from PLN (**E**) and ILN (**F**) are presented as the mean +/- SD for each group (*P < 0.05, **P < 0.01, ***P < 0.001).

In order to test our hypothesis that ipsilateral ILN and PLN collapse simultaneously, we first analyzed the B-in population of ipsilateral PLN and ILN from WT, two-month-old TNF-Tg mice prior to the onset of ankle arthritis, and TNF-Tg mice with bilateral ankle and asymmetric knee arthritis (Figure 5). The flow cytometry results showed that both expanding and collapsed PLN and ILN have a similar three-fold increase in total B-in numbers vs. aged matched WT controls. Moreover, this increase was disease specific, as no differences in B-in numbers were detected between WT and pre-arthritic TNF-Tg PLN and ILN. Finally, we observed similar percentages of hematopoietic cell populations between ipsilateral PLN and ILN (multicolor flow for CD1d, CD3, CD4, CD5, CD8, CD11b, CD11c, CD19, CD24, CD25, CD80, CD86, CD69, CD93, IgD and GL7, data not shown), which is consistent with our previous findings [29]. Thus, B-in expansion in ipsilateral PLN and ILN occurs simultaneously.

### B cell translocation into LYVE-1+ sinuses in collapsed ipsilateral PLN and ILN

**Figure 6:**
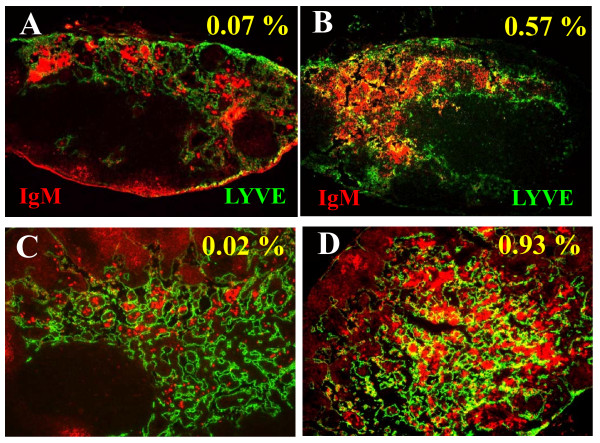
**Ipsilateral PLN and ILN collapse is associated with B cell translocation into LYVE+ sinuses**. Ipsilateral pairs of PLN and ILN (*n *= 4) from the mice described in Figure 1 were processed for IHC with fluorescently labeled antibodies against IgM and LYVE-1 to image the B cells (red) and lymphatic endothelium (green) respectively. Multicolor fluorescent micrographs (5x) of representative ILN (**A, B**) and PLN (**C, D**) are presented to illustrate the distinct staining in expanding nodes (A, C), versus the apparent co-localization (yellow) in the collapsed nodes (B, D), due to the B cells that have translocated into the paracortical lymphatic sinuses. The images were analyzed in Image-Pro Plus and the percentage of yellow pixels representing the overlapping signal is indicated.

Previously, we showed that expanding and collapsed PLN display distinct lymphoid architecture [29]. Expanding PLN have normal B cell follicles and T cell zone, with dilated paracortical sinuses filled with lymph that are mostly free of cells, suggesting active draining function. In collapsed PLN, the architecture of the B cell follicles and T cell zone are totally disrupted by B-in translocation into the paracortical sinuses in the center of the node, consistent with decreased draining function. As this B-in translocation and decreased interstitial space within the lymphatic vessels are the prominent histological differences between expanding and collapsed PLN, we investigated these features in ipsilateral PLN and ILN. Tissue sections were immunostained for both B cells and lymphatic endothelial cells with labeled antibodies against IgM and LYVE-1 respectively. Selective imaging of the IgM^hi ^cells, which includes the B-in population, was performed by signal intensity thresholding, and subsequent co-localization within lymphatic endothelium was assessed by superimposition two-color fluorescence microscopy images (Figure 6). The results demonstrated consistent association: all of expanding PLN were ipsilateral to ILN with wide lymphatic vessels that were void of IgM^hi ^cells (< 0.1% overlap with LYVE-1). Conversely, all of the collapsed PLN were ipsilateral to ILN whose lymphatic vessels were filled with IgM^hi ^cells. These IHC results support the hypothesis that asymmetric knee flare is mediated by simultaneous ipsilateral ILN and PLN collapse due to the translocation of B-in to the lumen of the lymphatic vessels of the nodes. We predict that these translocated B-in cells obstruct the lymphatics of the lower limb resulting in decreased afferent flow from the knee to the ILN.

### Afferent lymphatic flow to ipsilateral lymph nodes is associated with knee synovitis

**Figure 7:**
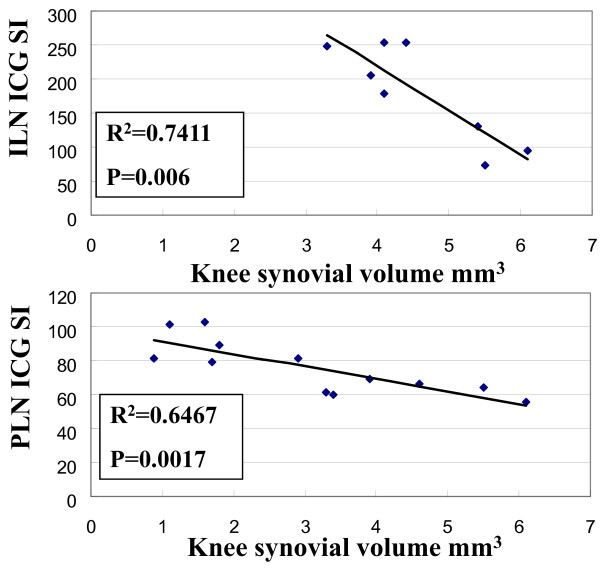
**Decreased lymphatic flow to ipsilateral PLN and ILN correlates with increased knee synovitis**. The knee synovial volume of TNF-Tg mice (ages three to less than nine months) at different stages of disease was quantified by CE-MRI. The lymphatic draining function of PLN and ILN in these mice was quantified independently by ICG-NIR imaging 30 minutes and 10 minutes after injection respectively by determining the signal intensity (SI) of the node. These data were used to assess the direct relationship between knee synovitis and lymph draining function in the ipsilateral ILN and PLN via linear regression analyses, in which the correlation coefficient (R^2^) with its statistical significance (P) is shown.

To assess the direct association between knee synovitis and afferent lymphatic flow to ipsilateral lymph nodes, a cohort of TNF-Tg mice with a broad range of knee arthritis was identified by performing CE-MRI on animals three to less than nine months of age to quantify knee synovial volume. Subsequently, ICG-NIR imaging was performed to quantify the signal intensity of PLN or ILN independently. Linear regression analysis of these data revealed highly significant correlations (Figure 7). These results support a model in which approximately 70% of knee flare in TNF-Tg mice can be explained by decreased lymphatic flow.

## Discussion

Our understanding of the events that lead to joint flare is incomplete, and critical questions remain answered. Specifically, the mechanisms that promote the development of asymmetric arthritis in the setting of a systemic immune mediated inflammatory disease have yet to be identified. We have previously demonstrated that alterations in PLN correlate with knee flare in TNF-Tg mice [22,28,29]. These intriguing observations provoked us to interrogate this potential biomarker, which predictably increases in size and contrast enhancement during a prolonged expansion phase, followed by a sudden collapse (Figure 1). Since these descriptive phenotypes are determined by a quantitative outcome measure, we set out to find an empirical CE-MRI threshold value that can objectively segregate expanding vs. collapsed PLN. Here we demonstrate this value to be LNcap = 30 (Table 1). Interestingly, LNCE = 5 also proved to be a reliable threshold value, while LN volume did not, demonstrating the importance of perfusion over size in this biomarker of arthritic flare.

The observation that asymmetric PLN collapse occurs concomitantly with arthritic flare in the adjacent knee (Figure 2), leads to the prediction that there also must be asymmetry in lymphatic draining function in the lower limb. Our ICG-NIR imaging results demonstrate this to be true (Figure 3), and support our conclusion from the CE-MRI data that the functional significance of the PLN in the arthritic flare process in the adjacent knee is dependent on lymphatic flow and not on node size.

The importance of lymphatic flow is also underscored by the fact that there are no significant cellular differences between expanding and collapsed PLN as determined by assessment of surface markers, proliferation and B cell heterogeneity, although they both have a significantly increased B-in population [29]. However, we did observe a histological difference between these phenotypes in that expanding PLN contain large paracortical sinuses devoid of IgM+ cells, while in collapsed PLN the paracortical sinuses were filled with IgM+ cells. This tissue morphology is consistent with "clogging" of lymphatic vessels by B cell aggregates and resultant, diminished lymphatic flow as observed in collapsed vs. expanding PLN (Figure 2).

The biggest surprise of this study was the finding that the ILN drains the knee (Figure 4), which initially appeared to be inconsistent with a model where collapse of PLN-induces an arthritic flare in the ipsilateral knee. One potential explanation is that ipsilateral PLN and ILN collapse is triggered by the same stimuli and occurs simultaneously. In support of this theory, we found that B-in expansion (Figure 5) and translocation (Figure 6) also occur simultaneously in ipsilateral PLN and ILN. Moreover, we found that lymphatic drainage to both PLN and ILN significantly correlate with knee synovial volume in TNF-Tg mice (Figure 7), suggesting that a single mechanism may be responsible for LN collapse in the same limb. It is important to note that these experiments were limited by the facts that PLN drain to ILN sequentially, thus making quantification of lymphatic flow to ipsilateral PLN and ILN impossible; and that ICG-NIR imaging of ILN requires euthanasia to expose the abdominal cavity, which limits quantitative assessment to a single time point. Nevertheless, the linkage of PLN and ILN collapse with the onset of ipsilateral knee synovitis strongly supports the existence of regional lymphatic factors that mediate joint flare during chronic inflammatory arthritis. Although purely speculative at this time, we find that this experimental evidence points to a central neuromuscular cascade that innervates the lymphatics along the axial plane of the limb, and dominates the local intrinsic lymphatic pumps that are known to be under adrenergic, cholinergic and peptinergic control [35]. Experiments to elucidate this central neuromuscular signal are ongoing.

The emerging paradigm to explain the pathogenesis of inflammatory arthritis posits that the disease initiates in the small distal joints of the flanges as a tenosynovitis, which rapidly spreads to the adjacent joint due to the immediate proximity of the inflamed synovial sheath and the synovium [30]. The chronic inflammation in these small joints stimulates lymphangiogenesis to limit the progression of synovitis and pannus formation by removing the immune cells and catabolic factors. Thus, disease spreads to the large-proximal joints only when the lymphatic drainage capacity of the limb is severely impaired, or a yet to be identified incident triggers LN collapse. Here we provide the first evidence that LN collapse occurs in series along an ipsilateral axis. The potential clinical significance of this is that the underappreciated enlarged efferent LN of RA joints that are often palpable on exam, or evident on imaging studies, may reflect disease activity and potentially a response to therapy. To explore this possibility we are currently evaluating the potential of MRI and ultrasound imaging to phenotype LN in RA patients as expanding or collapsed (ClinicalTrials.gov ID# NCT01098201, NCT01083563). Moreover, this model predicts that at least a component of the efficacy of BCDT is derived from its ability to clear B-in from lymphatic endothelium and thus "unclog" the sinuses and restore lymphatic flow. Certainly this hypothesis is testable in animal models and clinical trials, and future studies will determine the overall importance of this process in the etiology of arthritic flare.

## Conclusions

Asymmetric knee arthritis in TNF-Tg mice is triggered by simultaneous collapse of ipsilateral PLN and ILN, which is likely due to B-in translocation to the lumen of lymphatic vessels of both lymph nodes and result in a dramatic decrease in afferent lymphatic flow in the lower limb. As PLN and ILN function in series, PLN function serves as a new biomarker of arthritic flare in the adjacent knee.

## Abbreviations

AU: arbitrary unit; B-in: B cells in inflamed node; CE-MRI: contrast-enhanced magnetic resonance imaging; ICG-NIR: indocyanine green near-infrared; ILN: iliac lymph node; LN cap: lymph node capacity; LN CE: lymph node contrast enhancement; LN vol: lymph node volume; PLN: popliteal lymph node; RA: rheumatoid arthritis; TNF-Tg: tumor necrosis factor transgenic.

## Competing interests

The authors declare that they have no competing interests.

## Authors' contributions

JL performed most of the experiments, analyzed the data and participated in the manuscript draft. QZ participated in part of the ICG-NIR lymphatics imaging and data analysis. IK helped with flow cytometry. RWW, AB, LX and CTR provided scientific input and helped with manuscript editing. EMS designed the study, and drafted and finalized the manuscript. All authors read and approved the final manuscript.
